# Surgical Management of Posttraumatic Breast Bisection Following Seat Belt Injury: A Series of 3 Cases and Technique Description

**DOI:** 10.1093/asjof/ojag107

**Published:** 2026-06-08

**Authors:** Alexandre Bussy, Paul Frobert, Richard Vaucher, Simon Perez, Franck Dupuy, Emmanuel Delay

## Abstract

Posttraumatic breast bisection secondary to seat belt injury is a rare and often unrecognized entity that may mimic breast cancer. It results from the formation of a transfixing fibrotic scar band responsible for a sharp linear deformity of the breast. Few cases have been reported in the literature, most often as isolated case reports. We report a single-center retrospective series of 3 patients who underwent surgical management in our institution for posttraumatic breast deformity following seat belt injury. Clinical, radiological, operative, histopathological, and follow-up data were analyzed. All patients presented with a transfixing fibrotic band deformity associated with imaging-confirmed fat necrosis. The delay between trauma and surgical consultation ranged from 3 to 9 months. Surgical management consisted of bilateral reduction mammoplasty with complete excision of the fibrotic tissue. Glandular reconstruction relied on the use of a posterior glandular flap, combined with lipomodeling. Postoperative courses were uneventful. Follow-up exceeded 12 months for all patients, with stable and satisfactory aesthetic outcomes and no recurrence of deformity. Posttraumatic breast bisection must be recognized to avoid confusion with breast malignancy and to ensure appropriate management. Surgical treatment is based on excision of fibrotic tissue and glandular reshaping. The posterior glandular flap, combined when necessary with lipomodeling, represents a simple, reproducible, and effective technique, providing durable aesthetic results.

**Level of Evidence**: 4 (Therapeutic) 
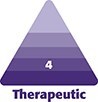

Seat belts have significantly reduced mortality and severe injuries related to road traffic accidents. Their use is now mandatory, with compliance rates close to 100% in France. This evolution has nevertheless been associated with the emergence of specific injuries grouped under the term seat belt injuries. The literature mainly describes rib fractures and potentially severe thoracic hemorrhages,^1^ as well as soft-tissue defects of the chest wall.^2^ Direct breast injuries are rarely reported,^3-5^ although they may lead to the characteristic appearance of a breast bisection remote from the initial trauma.

Management in the acute phase depends on the severity of breast injuries and ranges from compressive dressings,^6^ to vascular embolization,^7-9^ or even radical hemostatic surgical procedures such as emergency mastectomy.^10,11^ In patients with silicone implants, prosthetic rupture^12^ or secondary capsular contracture has also been reported.

In 2007, Majeski et al^13^ proposed an initial classification of breast trauma into 4 stages limited to immediate injuries, ranging from simple bruising to breast avulsion. More recently, in 2014, Song and Teo^14^ published a PRISMA-based systematic review and proposed a 4-stage classification:

- Stage 1a: Immediate pain and bruising, sometimes with asymmetry. Initial imaging is recommended to rule out rib fracture or implant rupture.
- Stage 1b: Rapid increase in breast volume, most often related to active hemorrhage, requiring urgent management.
- Stage 2a: Delayed presentation as a palpable mass, most often related to fat necrosis, justifying triple assessment (clinical, radiological, and histological).
- Stage 2b: Late aesthetic deformities such as fibrotic retraction or breast bisection deformity; triple assessment should be considered.

Acute trauma tends to heal spontaneously, but hematoma formation and tissue crushing promote fat necrosis, oil cyst formation, and subsequent fibrotic bands responsible for secondary breast deformities.^15-17^ Clinical examination of an affected breast (stage 2b) typically reveals asymmetry, cicatricial retraction, and sometimes a “peau d’orange” appearance related to lymphatic and fibrotic distortion.^18,19^ Fat necrosis is reported in nearly 70% of compression-related breast trauma cases.^20^

Reported cases in the literature are mostly isolated observations or very small series. To our knowledge, no publication has described more than 2 patients managed in the same center. The present study therefore represents the largest published series to date and provides a detailed analysis of the surgical management of these posttraumatic breast deformities.

The aim of this study is to update the management of posttraumatic breast bisection secondary to seat belt trauma, based on 3 clinical cases treated by our team and a review of recent literature.

## METHODS

We conducted a single-center retrospective study in our department of plastic and reconstructive surgery.

### Study Population

Three patients presenting with posttraumatic breast deformity secondary to seat belt injury were surgically treated over the past 20 years. No patients were excluded.

### Data Collection

Information was extracted from medical records, including:

- Demographic characteristics (age, medical history)
- Circumstances and delay of trauma
- Clinical presentation and breast examination
- Preoperative imaging (mammography, ultrasound, MRI when applicable)
- Surgical technique used (type of mammoplasty, glandular flap, lipomodeling, resection or injection volumes)
- Histopathological findings
- Postoperative course, clinical follow-up, and complications

All patients underwent a complete clinical examination and preoperative and postoperative photographic documentation.

### Preoperative Assessment

All patients underwent bilateral mammography and ultrasound. MRI was not required in our cases due to typical benign imaging features. Computed tomography (CT) was not performed for breast evaluation.

### Ethical Considerations

This retrospective study was conducted in accordance with French data protection regulations (MR-003, CNIL) and did not require formal Institutional Review Board approval. All patients were informed about the use of their medical data for research and educational purposes, and written informed consent was obtained for the use of clinical photographs. The study was conducted in accordance with the principles of the Declaration of Helsinki.

### Surgical Technique

In all 3 cases, bilateral breast reduction was performed. The technique was adapted to the location of the fibrotic band and breast volume, while respecting the following principles.

Preoperative skin markings were performed with the patient standing, including determination of the future position of the nipple–areola complex (NAC). The choice of areolar pedicle (superomedial or superolateral) depended on the location of fibrosis to avoid including it in the NAC-bearing flap. Surgery began with an inverted-T skin incision followed by de-epithelialization of the reduction areas. The fibrotic zone, often clinically visible as a bisection, was systematically identified and completely excised, along with any fat necrosis cysts. This resection usually resulted in significant glandular loss.

Breast reconstruction was achieved using a posterior glandular flap,^21^ pedicled on its posterior base and vascularized by perforators from the thoracoacromial artery and intercostal branches. This flap, measuring approximately 8 to 9 cm with an average weight of 150 g, was transposed into the defect and fixed to the pectoralis major muscle using resorbable 1 polyglactin sutures, with surrounding glandular reshaping to restore breast contour.

Additional lipomodeling was then performed by harvesting autologous fat, centrifugation (1000 rpm for 15 seconds), and reinjection in a multiplanar fashion, including subcutaneous and intraglandular injections within the pedicle pillars. Care was taken to avoid direct injection into the resection cavity to reduce the risk of fat necrosis and oil cyst formation, mainly targeting the upper quadrants and cleavage.^22^ Closure was performed in 3 layers with periareolar and inverted-T scars. Breast symmetry was checked in a semi-seated position, followed by application of a compressive dressing.

## PATIENTS

We report 3 patients presenting with posttraumatic breast bisection (stage 2b according to the Song and Teo classification), all surgically managed in our department by the same surgeon using the same operative strategy.

Although the exact timing of deformity onset was not systematically documented, patients reported noticing progressive breast asymmetry after resolution of the initial posttraumatic edema, suggesting that fibrotic remodeling becomes clinically apparent once acute swelling subsides.

All patients underwent bilateral mammography and targeted breast ultrasound. MRI was not considered necessary, as imaging findings were typical and did not raise suspicion of malignancy. Consequently, no preoperative biopsy was performed.

All resected specimens were systematically submitted for histopathological examination, which confirmed fibrosis and fat necrosis without evidence of malignancy.

No early or late postoperative complications occurred. Specifically, no cases of infection, hematoma, NAC necrosis, delayed wound healing, recurrent fat necrosis, or need for revision surgery were recorded during follow-up.

### Case 1

History: A 60-year-old woman presented 6 months after a road traffic accident with right breast trauma.

**Figure 1.**
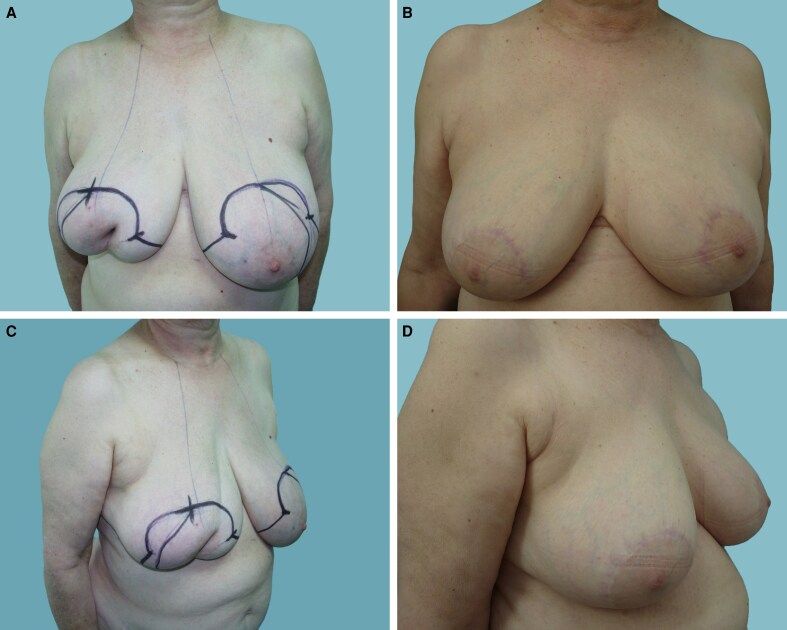
Case 1. A 60-year-old female presenting with posttraumatic breast bisection of the right breast following seat belt injury. (A, C) Preoperative view (frontal and oblique) showing breast asymmetry and glandular bisection. (B, D) Postoperative views (frontal and oblique) at 1 year postoperatively following bilateral reduction mammoplasty using a superomedial pedicle with excision of fibrotic tissue and fat necrosis (300 g resected on the right and 510 g on the left), combined with posterior glandular flap reconstruction and lipomodeling (280 mL injected into the right breast).

Clinical findings: Right breast glandular bisection caused by a transfixing fibrotic band, nipple inversion, and bilateral hypertrophy (Figure 1).

**Figure 2.**
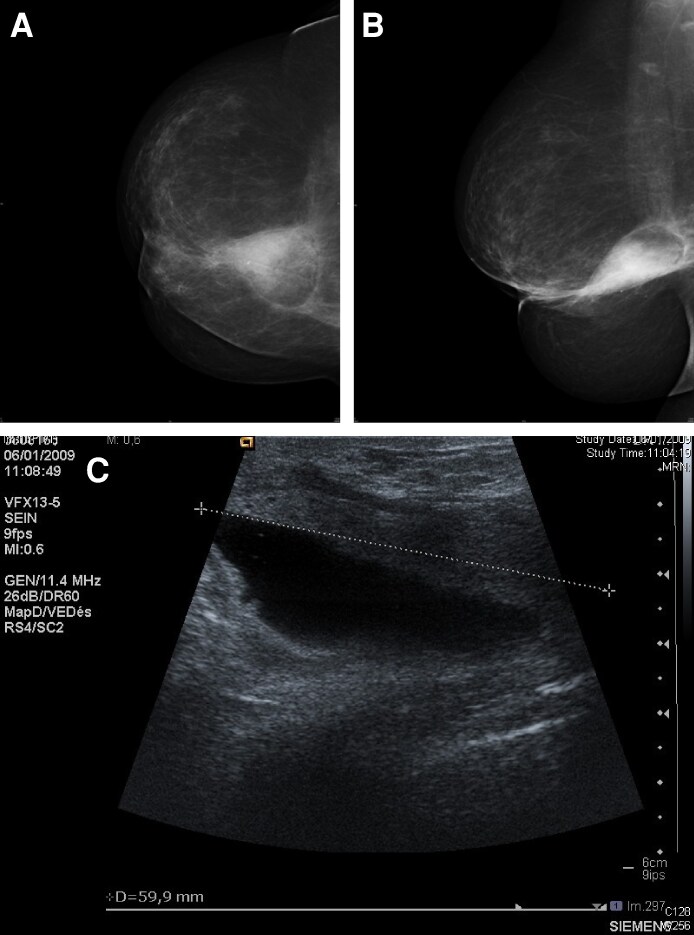
Imaging findings in a 60-year-old female with posttraumatic breast bisection following seat belt injury. (A, B) Mammography shows a well-defined radiolucent lesion with associated architectural distortion. (C) Ultrasound demonstrates a well-circumscribed anechoic cystic lesion with posterior acoustic enhancement, consistent with a typical fat necrosis (oil cyst).

Imaging: Ultrasound and mammography ruled out malignancy and revealed a fat necrosis cyst (Figure 2).

Management: Bilateral breast reduction (300 g resected on the right and 510 g on the left) with complete excision of fibrotic tissue and fat necrosis, using a superomedial NAC pedicle. Reconstruction was performed using a posterior glandular flap combined with complementary lipomodeling (280 mL injected into the right breast)

Histopathology: Fibrosis and fat necrosis without malignancy.

Follow-up: No early or late complications were observed. A stable aesthetic result was maintained at 12 months.

### Case 2

History: A 54-year-old woman was referred for bilateral breast hypertrophy with asymmetry. A road traffic accident occurring 9 months earlier was identified during clinical reassessment.

Clinical Findings: Initial examination in the standing position showed asymmetry. A fibrotic band was detected intraoperatively and confirmed in the supine position.

Imaging: Preoperative mammography and ultrasound were classified as ACR 2 (American College of Radiology category 2, benign finding).

Management: Bilateral breast reduction (750 g resected on the left and 670 g on the right) with excision of the fibrotic band and reconstruction using superomedial NAC pedicle combined with a posterior glandular flap and lipomodeling (250 mL injected into the right breast).

Histopathology: Fibrosis and fat necrosis without malignancy.

Follow-up: No early or late complications were observed. A stable aesthetic result was maintained at 12 months.

### Case 3

History: A 54-year-old woman sustained a road traffic accident 3 months earlier, complicated by a right breast hematoma that required repeated ultrasound-guided drainage.

**Figure 3.**
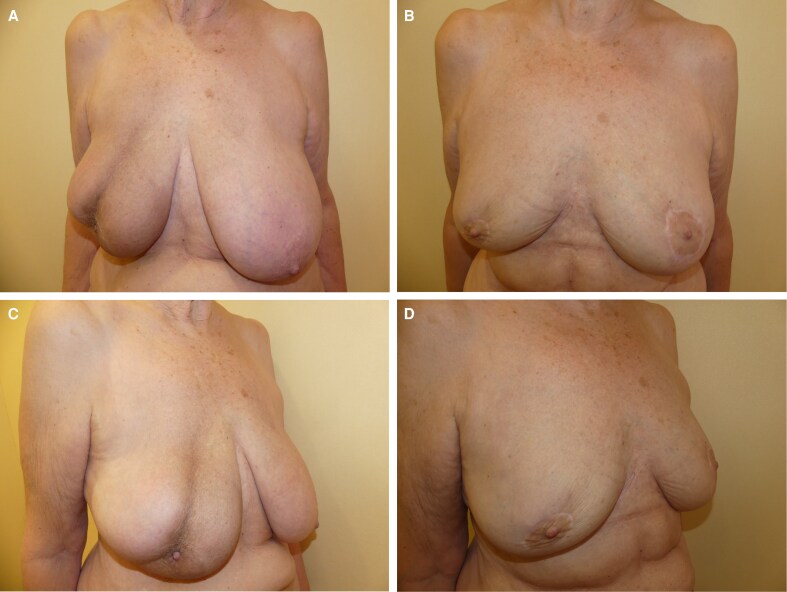
Case 3. A 54-year-old female presenting with posttraumatic breast bisection of the right breast. (A, C) Preoperative views (frontal and oblique) showing breast deformity and nipple inversion. (B, D) Postoperative views (frontal and oblique) at 6 months postoperatively following excision of fibrotic tissue and fat necrosis, reconstruction using a posterior glandular flap with a superolateral pedicle, and lipomodeling (205 mL injected into the right breast), combined with contralateral breast reduction for symmetrization (310 g resected).

Clinical Findings: Right breast hypertrophy with glandular bisection and nipple inversion (Figure 3).

Imaging: Ultrasound and mammography ruled out malignancy.

Management: Excision of fibrosis and fat necrosis was performed on the right breast. Reconstruction used a posterior glandular flap with a superolateral NAC pedicle, combined with complementary lipomodeling (205 mL injected into the right breast). A contralateral left breast reduction for symmetrization was performed (310 g resected). A supplementary video details the surgical steps (Video).

Histopathology: Fibrosis and fat necrosis without malignancy.

Follow-up: No early or late complications were observed. A stable aesthetic result was maintained at 12 months.

## DISCUSSION

Posttraumatic breast bisection secondary to seat belt injury is a rare entity. Most publications report isolated cases, with only a few very limited series described. Over the past 30 years, we identified 7 articles reporting surgical management of this deformity, summarized in Table 1. Most patients were middle-aged or older women presenting with a bisection deformity of a hypertrophic breast, often associated with marked ptosis. A single male case has been reported.^24^ Surgical techniques were heterogeneous, ranging from simple excision of the fibrotic band to reduction mammoplasty using dermoglandular flaps, sometimes combined with additional lipofilling.

**Table 1.** Published Surgical Management of Posttraumatic Breast Bisection Deformities; Breast Ptosis Was Graded According to the Regnault Classification

Author, year	Study design	Patient	Breast deformity	Surgical approach	Fat grafting
Mayer et al^17^	Case report	Female, 58 years	Left breast, medial quadrant retraction with areolar dystopia	Bilateral reduction mammoplasty with posterior glandular flap	No
Lafford et al^23^	Case report	Female, 63 years	Right breast, breast bisection deformity with NAC inversion, grade III ptosis	Bilateral superior pedicle reduction mammoplasty	No
Noel et al^24^	Case series	Female, 53 years	Right breast, breast bisection deformity without NAC involvement, grade III ptosis	Inferior pedicle reduction mammoplasty	No
		Male, 47 years	Left breast, adipomastia with muscle rupture and fat necrosis and Morel-Lavallée syndrome	Submammary incision with debridement	No
Petrie^16^	Case report	Female, 69 years	Right breast, breast bisection deformity with NAC inversion, grade II ptosis	Excision and suturing of invaginated area with NAC elevation	Yes
Teo et al^25^	Case report	Female, 67 years	Right breast, breast bisection deformity with NAC inversion, grade II ptosis	NAC elevation on superolateral pedicle with pillar reconstruction	No
Scevola et al^5^	Case series	Female, 54 years	Right breast, breast bisection deformity only in suppine position	Bilateral reduction mammoplasty with superomedial pedicle and posterior glandular flap and lipomodeling	Yes
		Female, 60 years	Right breast, breast bisection deformity with NAC inversion on hypertrophic breasts		Yes
Paddle et al^15^	Case report	Female, 37 years	Right breast, breast bisection deformity, hypertrophy, grade III ptosis	Reduction mammoplasty (modified Hall-Findlay technique) with en bloc excision^26^	No

### Differential Diagnosis and Preoperative Assessment

One of the main challenges of this pathology is differentiating it from breast cancer. Fibrotic scars and fat necrosis changes may mimic skin retraction or a suspicious mass. Systematic preoperative breast imaging is therefore mandatory.^19^ Mammography and ultrasound usually exclude most differential diagnoses, while MRI may be useful in atypical cases.^18^ In cases of persistent doubt, core needle biopsy remains necessary, as coexistence of breast carcinoma has been reported following breast trauma.^27,28^ No vascular compromise was clinically demonstrated in our delayed cases. Therefore, angiography is not indicated preoperatively, outside acute hemorrhagic settings.

In one case, the fibrotic band was not clearly identified during preoperative examination in the standing position and became apparent only intraoperatively in the supine position. This likely reflects changes in tissue tension and breast redistribution. This observation underscores the importance of systematic examination in both standing and supine positions during preoperative assessment, particularly in patients with a history of seat belt trauma, as subtle fibrotic retraction may otherwise remain undetected.

### Timing of Corrective Surgery

The timing of corrective surgery remains variable in the literature. Reported delays between trauma and surgical management range from 2 to 12 months in most cases, although very late presentations have also been described, including several years after the initial injury. In our series, patients were operated between 3 and 9 months after trauma.

Posttraumatic fibrosis likely reflects a healing response to glandular disruption and may therefore be difficult to prevent once the initial injury has occurred. In the acute phase, edema and hematoma may mask underlying structural damage, delaying clinical recognition.

Surgical intervention should preferably be delayed until acute inflammatory changes and posttraumatic edema have resolved and fibrotic remodeling has stabilized. Operating too early may expose patients to evolving tissue changes and unpredictable contour remodeling. Therefore, corrective surgery is best considered once breast asymmetry has become clinically stable and malignancy has been excluded through appropriate imaging.

### Reported Surgical Strategies

Surgical techniques described in the literature vary and should be tailored to the severity of the deformity. Following less traumatic seat belt injuries, patients may develop partial fibrotic retraction or subtle contour irregularities without complete glandular bisection. In such cases, conservative management or isolated lipofilling may be sufficient. For small but well-defined fibrotic bands, limited excision can be considered, although it carries a risk of recurrence and residual asymmetry. In contrast, severe deformities characterized by complete glandular bisection and significant fibrosis generally require reduction mammoplasty techniques using dermoglandular flaps to restore breast shape. However, when extensive fibrotic excision is necessary, the resulting deep glandular defect may create a substantial volume loss that must be adequately addressed to achieve durable contour restoration.^22,29^

In this context, our experience highlights the value of the posterior glandular flap. Well vascularized by thoracoacromial and intercostal perforators,^30^ it effectively fills the defect after complete excision of fibrosis. Combined with lipomodeling, it further improves outcomes, particularly in the upper quadrants and cleavage, which are difficult to correct with glandular reshaping alone. A second session may sometimes be indicated to optimize aesthetic results.

### Contribution of Our Series

Our 3 cases demonstrate the reproducibility of this technique. Despite slightly different clinical presentations, the same surgical strategy was applied, yielding satisfactory and stable aesthetic results. A major strength of our series is long-term follow-up: all patients were reviewed more than 12 months postoperatively. In all cases, aesthetic results were maintained without recurrence, which is significant given the rarity of this condition.

### Limitations and Perspectives

**Figure 4.**
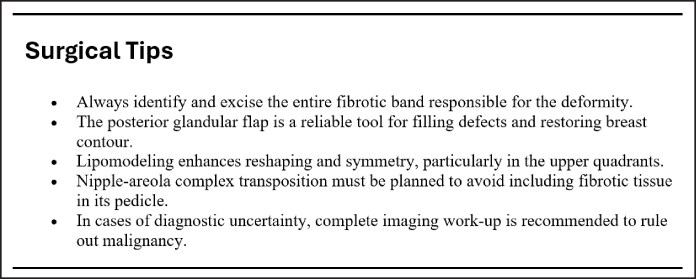
Surgical tips for the management of posttraumatic breast bisection following seat belt injury.

The limitations of this study include the small sample size (*n* = 3), its retrospective design, and the absence of objective outcome measures and validated patient-reported outcome measures, which may restrict the ability to quantitatively assess aesthetic and patient-centered outcomes. However, given the exceptional nature of this pathology, larger series are unlikely to be rapidly established. Sharing structured surgical principles and clinical experience between centers may help refine management strategies over time. This report aims to provide a practical surgical framework for surgeons confronted with these rare cases. The supplementary video illustrates the key technical steps, and a set of surgical tips outlining the main components of patient management is provided (Figure 4).

## CONCLUSIONS

Posttraumatic breast bisection is a rare but distinctive sequela of seat belt injury that requires careful diagnostic evaluation to exclude malignancy. Our series illustrates a consistent surgical strategy combining complete excision of fibrotic tissue, reduction mammoplasty, and posterior glandular flap reconstruction, optionally supplemented with lipomodeling. This approach provided stable aesthetic outcomes without complications in all cases. Although management must remain individualized, these principles may serve as a practical framework for surgeons confronted with similar deformities.

## Supplemental Material

This article contains supplemental material located online at https://doi.org/10.1093/asjof/ojag107.

## Supplementary Material

ojag107_Supplementary_Data
