# Supplementary figures and images for: Surgical Management of Posttraumatic Breast Bisection Following Seat Belt Injury: A Series of 3 Cases and Technique Description
Source: Aesthet Surg J Open Forum. 2026 Jun 8;8:ojag107. doi: 10.1093/asjof/ojag107 (PMC13418189; doi:10.1093/asjof/ojag107)

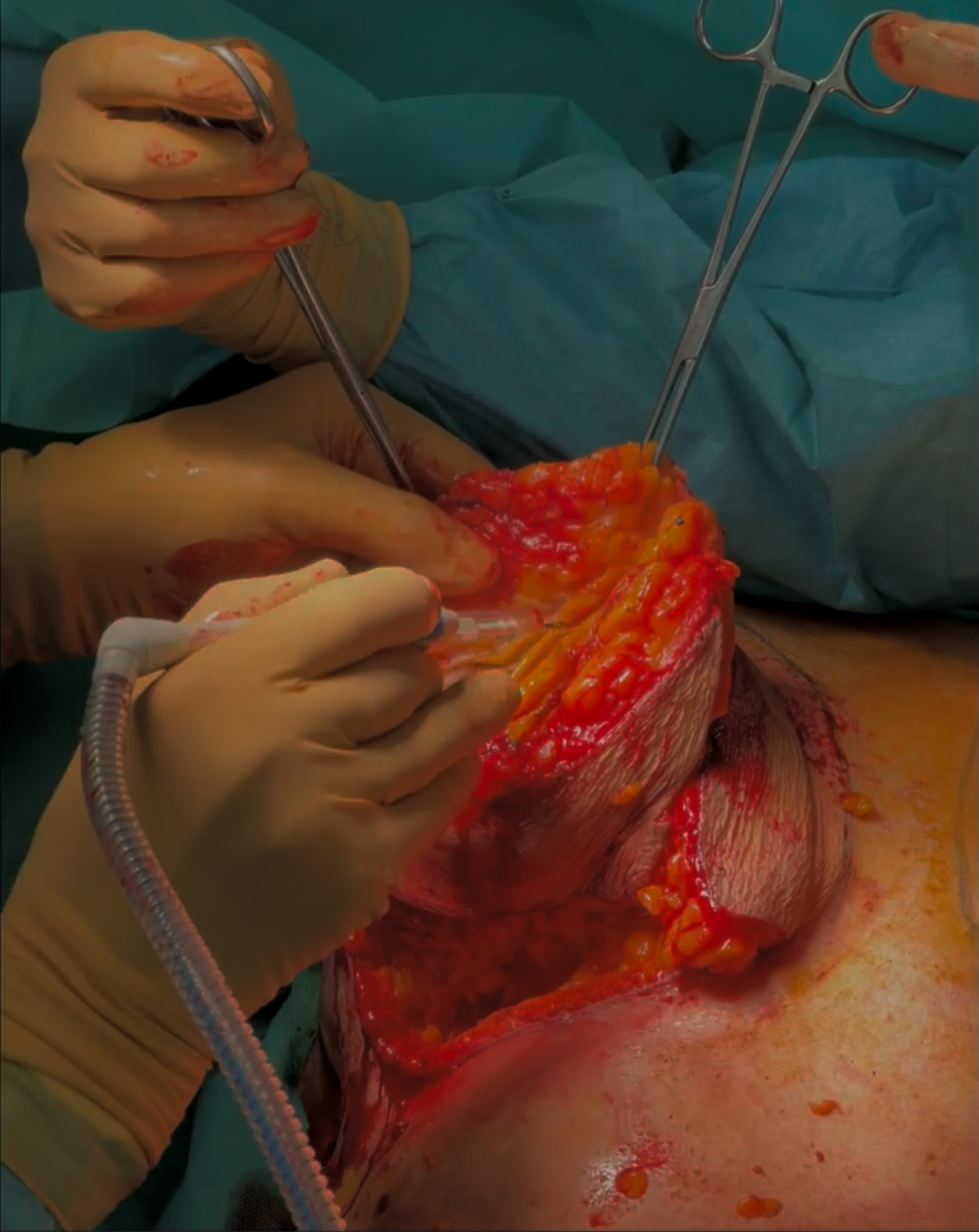

Supplement: ojag107_Supplementary_Data [file ojag107_Supplementary_Data.zip › Video still.png]
